# Associations of cognitive dysfunction with motor and non-motor symptoms in patients with de novo Parkinson’s disease

**DOI:** 10.1038/s41598-022-15630-8

**Published:** 2022-07-06

**Authors:** Kyum-Yil Kwon, Suyeon Park, Rae On Kim, Eun Ji Lee, Mina Lee

**Affiliations:** 1 Department of Neurology, Soonchunhyang University Seoul Hospital, Soonchunhyang University School of Medicine, 59 Daesagwan-ro, Yongsan-gu, Seoul, 04401 Republic of Korea; 2 Department of Biostatistics, Soonchunhyang University Seoul Hospital, Soonchunhyang University School of Medicine, Seoul, Republic of Korea; 3 Department of Applied Statistics, Chung-Ang University, Seoul, Republic of Korea

**Keywords:** Neuroscience, Diseases, Neurology

## Abstract

The risk factors of mild cognitive impairment (MCI) in patients with de novo Parkinson’s disease (PD) remain unclear. Therefore, the objective of this study was to compare motor and non-motor symptoms between de novo patients with PD with and without MCI. Moreover, detailed relationships between each cognitive deficit and other clinical characteristics in de novo patients with PD were investigated. Consecutive patients with de novo PD were retrospectively enrolled in this study. Motor symptoms were assessed using the Unified Parkinson’s Disease Rating Scale (UPDRS) part-III and the Hoehn and Yahr (HY) stage. Non-motor symptoms including depression, anxiety, fatigue, and autonomic dysfunction were measured using representative questionnaires. Motor symptoms, depression, and dysautonomia were associated with MCI in de novo patients with PD. Compared with the non-MCI group with PD, the MCI group with PD had higher scores of UPDRS-III, HY stage, depression, and dysautonomia, but not fatigue or anxiety. Both UPDRS-III and HY stage were significantly linked to all cognitive deficits except attention. Logistic regression analysis showed that depression was associated with memory, visuospatial, and executive impairment, and dysautonomia was related to visuospatial and executive impairment. The results of this study suggest that cognitive impairment in PD might have a different relationship pattern to the motor and some non-motor symptoms.

## Introduction

Neurodegenerative progression in Parkinson’s disease (PD) involves not only dopaminergic neurons, but also non-dopaminergic neurons including serotonergic, noradrenergic, and cholinergic neurons^1^. Besides motor symptoms-related disability, patients with PD frequently suffer from various non-motor symptoms including constipation, sleep disturbance, pain, and cognitive decline. Non-motor symptoms in PD are known to be heterogeneous since multi-systemic neurodegeneration and related neuropsychological changes are not constant^2^. Especially, cognitive impairment is one of the major factors that lower the quality of life in patients with PD^3,4^. Among the five cognitive domains, the pattern of the cognitive deficit varies across individuals even at the early stages of PD. Frontal-executive impairments in early PD are associated with prefrontal dopaminergic activity while posterior cortical dysfunctions appear to be non-dopaminergic deficits^5^. Kehagia et al. introduced the concept of the “dual syndrome hypothesis” in the cognitive decline of PD. That is, the dopaminergic circuits-related deficit is related to mild cognitive impairment (MCI) in PD, while the cholinergic circuits-associated deficit is linked to dementia in PD^6,7^. Such different cognitive deficits in PD imply that neurodegenerative progression might be different among patients with PD.

Studies on the relationship between cognitive impairment and other clinical characteristics in patients with PD have provided pathophysiological evidence for disease progression. A recent meta-analysis has shown that MCI in PD is significantly associated with various clinical characteristics including age, disease duration, disease severity, motor subtype, and non-motor symptoms such as apathy and depression^8^. However, to the best of our knowledge, none have revealed the association of specific cognitive dysfunction with clinical characteristics of patients with PD yet. Therefore, the objective of this study was to investigate the clinical features associated with MCI in patients with PD and examine detailed relationships of each cognitive deficit with other clinical characteristics in de novo patients with PD.

## Results

### Comparison of clinical characteristics between patients with PD with and without MCI

The patients’ baseline demographics and motor symptoms are described in Table 1. There were no differences in demographic features between de novo patients with PD with and without MCI. However, thirty-four patients with de novo PD with MCI (PD-MCI) exhibited higher scores in rigidity (*p* = 0.023), UPDRS-III (*p* = 0.024), and HY stage (*p* = 0.021) than 26 patients with de novo PD without MCI (non-PD-MCI). Other motor subscores were not significantly different between the two groups.

**Table 1:** Demographics and motor symptoms in de novo Parkinsonian patients with or without MCI.

Variable	MCI (n = 34)	Non-MCI (n = 26)	P value
Age (yr)	71.0 ± 8.6	68.9 ± 10.6	0.390
Sex-female	18 (52.9%)	15 (57.7%)	0.714
Body mass index	23.3 ± 3.1	24.1 ± 3.1	0.353
Level of education (yr)	10.4 ± 5.3	9.6 ± 4.5	0.534
Disease duration at registration (yr)	1.4 ± 1.1	1.4 ± 1.0	0.925
Follow-up duration (yr)	3.0 ± 1.0	2.8 ± 0.8	0.381
Hypertension	13 (38.24%)	13 (50%)	0.517
Diabetes	9 (26.47%)	6 (23.08%)	> 0.99
**UPDRS-III (motor)**			
Tremor score	3 (1, 4)	3 (1–4)	0.769^a^
Rigidity score	7 (2.5, 8)	3.5 (2–7)	**0.023**^a^
Bradykinesia score	13.4 ± 5.59	10.5 ± 5.5	0.056
PIGD score	2 (1, 5.75)	2 (1–3)	0.137^a^
Total motor score	28.4 ± 11.4	21.7 ± 10.3	**0.024**
**Motor subtype**			0.252
Tremor dominant	12 (35.3%)	13 (50%)	
Non-tremor dominant	22 (64.7%)	13 (50%)	
HY stage	2 (2, 2.5)	2 (1.6, 2)	**0.021**^a^

Data are presented as *n* (%) for categorical variables or mean ± S.D./median (interquartile range) values for continuous variables.

MCI, mild cognitive impairment; UPDRS, Unified Parkinson’s disease rating scale; PIGD, postural instability and gait difficulty; HY, Hoehn and Yahr.

Statistical analyses were conducted with Chi square test for categorical variables, Student t-test or Mann–Whitney U test ^a^for continuous variables.

Significant values are in bold.

Comparisons of cognition and other non-motor symptoms between PD-MCI and non-PD-MCI groups are shown in Table 2. As expected, all cognitive functions except attention were significantly decreased in the PD-MCI group than in the non-PD-MCI group. For other non-motor symptoms, the PD-MCI group not only showed more depression (*p* = 0.0151), but also showed more severe total dysautonomia (*p* < 0.001) than the non-PD-MCI group. Specifically, scores of gastrointestinal (GI) (*p* = 0.003), urinary (UR) (*p* = 0.005), cardiovascular (CV) (*p* = 0.005), and thermoregulatory (TR) (*p* = 0.047) domains were higher for the PD-MCI group than for the non-PD-MCI group. However, scores for anxiety, fatigue, and pilomotor (PM) domain of dysautonomia were not significantly different between the two groups.

**Table 2:** Comparison of cognition and other non-motor symptoms in de novo Parkinsonian patients with or without MCI.

Variable	MCI	Non-MCI	P value
K-MMSE, score	24.4 ± 4.4	27.3 ± 2.1	**0.0015**
**Cognitive subdomain (composite %ile)**			
Attention and working memory	79.7 ± 21.3	80.0 ± 19.3	0.945
Language	41.4 ± 33.0	68.6 ± 19.0	** < 0.001**
Memory	13.3 ± 19.1	47.8 ± 23.9	** < 0.001**
Visuospatial function	32.5 ± 29.6	59.7 ± 30.0	** < 0.001**
Executive function	18.5 ± 21.0	54.7 ± 26.3	** < 0.001**
BDI (depression)	12.9 ± 9.1	6.9 ± 5.1	**0.015**
BAI (anxiety)	8.0 ± 6.8	5.9 ± 6.1	0.380
PFS (fatigue)	45.7 ± 17.6	38.2 ± 14.9	0.473
**SCOPA-AUT (dysautonomia)**			
Gastrointestinal (GI) domain	4.5 (2–7)	1 (0–3)	**0.003**^a^
Urinary (UR) domain	8.5 (5.25–11.75)	4 (2.25–6)	**0.005**^a^
Cardiovascular (CV) domain	1 (0–2)	0 (0–0)	**0.005**^a^
Thermoregulatory (TR) domain	1 (0–2)	0 (0–0.75)	**0.047**^a^
Pilomotor (PM) domain	0 (0–0)	0 (0–0)	0.520^a^
Total dysautonomia	17.1 ± 8.4	9.0 ± 6.5	** < 0.001**

Data are shown as mean ± S.D., or median (interquartile range) values.

MCI, mild cognitive impairment; K-MMSE, Korean version of mini-mental state examination; BDI, Beck depression inventory; BAI, Beck anxiety inventory; PFS, Parkinson’s disease fatigue scale; SCOPA-AUT, the Scale for Outcomes in Parkinson’s disease-Autonomic questionnaire.

Statistical analyses were conducted with Chi square test for categorical variables, and Student t-test or Mann–Whitney U test ^a^for continuous variables.

Significant values are in bold.

### Comparison of motor or non-motor symptoms between de novo PD patients with and without impairment in each cognitive domain

**Figure 1:**
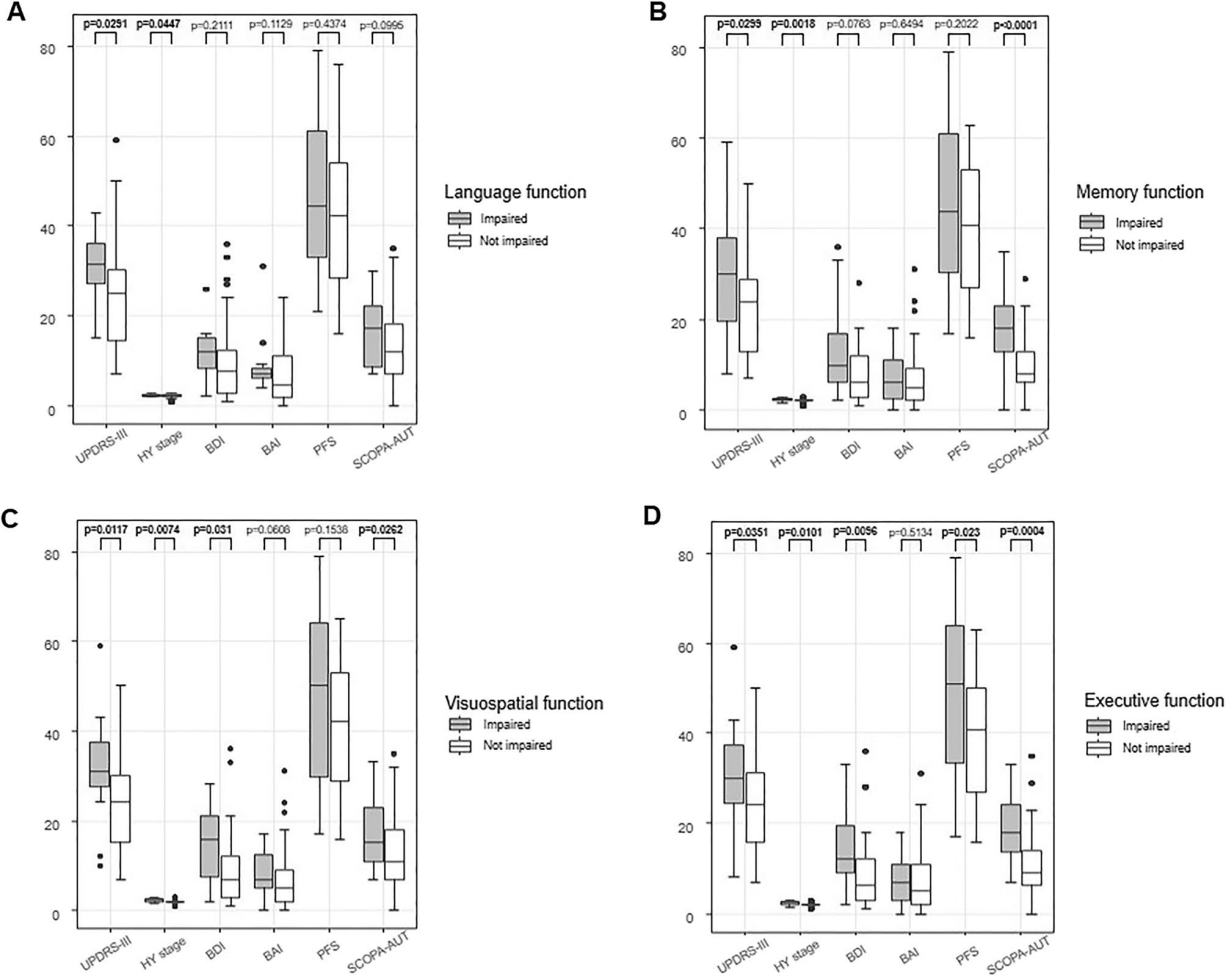
Comparison of motor or non-motor symptoms between de novo PD patients with and without impairment in each cognitive domain. Comparison of motor or non-motor symptoms between patients with de novo PD with and without language impairment (**A**). Comparison of motor or non-motor symptoms between those with and without memory impairment (**B**). Comparison of motor or non-motor symptoms between those with and without visuospatial impairment (**C**). Comparison of motor or non-motor symptoms between those with and without executive impairment (**D**). Y-axes indicate scores for each variable including UPDRS-III, HY stage, BDI, BAI, PFS, or SCOPA-AUT in the x-axes, thereby the units of the y-axes are dependent on the clinical variables in the x-axes. Abbreviations: PD, Parkinson’s disease; UPDRS-III, Unified Parkinson’s disease rating scale-part 3; HY stage, Hoehn and Yahr stage; BDI, Beck depression inventory; BAI, Beck anxiety inventory; PFS, Parkinson’s disease fatigue scale; SCOPA-AUT, the Scale for Outcomes in Parkinson’s disease-Autonomic questionnaire.

Comparisons of motor or non-motor features in de novo patients with PD with and without impairment in each cognitive domain were performed in detail (Fig. 1, Supplementary Table 1, and Supplementary Fig. 1). In our study, the attention-working memory domain was not impaired in any subject in our study population.

For the language domain as shown in Fig. 1A, compared with 42 patients without language impairment, 12 patients with language impairment had higher scores of total motor symptom [median (IQR) of impaired vs. non-impaired: 31.5 (27.0–36.25) vs. 25 (14.5–30.25); *p* = 0.0291] and HY stage [median (IQR) of impaired vs. non-impaired: 2.25 (2–2.5) vs. 2 (2–2.5); *p* = 0.0447], while the scores of depression, anxiety, fatigue, and total dysautonomia were not significantly different between the two groups.

For memory domain as shown in Fig. 1B, compared with 33 patients without memory impairment, 27 patients with memory impairment exhibited higher scores of total motor symptom [median (IQR) of impaired vs. non-impaired: 30 (19.5–38) vs. 24 (13.0–29); *p* = 0.0291], HY stage [median (IQR) of impaired vs. non-impaired: 2.5 (2–2.5) vs. 2.0 (2–2.0); *p* = 0.0018], and total dysautonomia [median (IQR) of impaired vs. non-impaired: 18 (13–23) vs. 8 (6–13); *p* < 0.0001], while scores of depression, anxiety, and fatigue were not significantly different between the two groups.

For visuospatial function as shown in Fig. 1C, compared with 45 patients without visuospatial impairment, 15 patients with visuospatial impairment showed higher scores of total motor symptom [median (IQR) of impaired vs. non-impaired: 31 (27.5–37.5) vs. 24 (15.0–30.0); *p* = 0.0117], HY stage [median (IQR) of impaired vs. non-impaired: 2.5 (2–2.5) vs. 2.0 (2–2.0); *p* = 0.0074], depression [median (IQR) of impaired vs. non-impaired: 16 (7.5–21) vs. 7 (3.0–12); *p* = 0.031], and total dysautonomia [median (IQR) of impaired vs. non-impaired: 15 (11–23) vs. 11 (7–18); *p* = 0.0262], while scores of anxiety and fatigue were not significantly different between the two groups.

For executive function as shown in Fig. 1D, compared with 41 patients without executive impairment, 19 patients with executive impairment had higher scores of total motor symptom [median (IQR) of impaired vs. non-impaired: 30 (24.5–37.5) vs. 24 (16.0–31.0); *p* = 0.0351], HY stage [median (IQR) of impaired vs. non-impaired: 2.5 (2–2.5) vs. 2.0 (2–2.0); *p* = 0.0101], depression [median (IQR) of impaired vs. non-impaired: 12 (9–19.5) vs. 6 (3–12.0); *p* = 0.0091], fatigue [median (IQR) of impaired vs. non-impaired: 51 (33.5–64) vs. 41 (27.0–50); *p* = 0.023], and total dysautonomia [median (IQR) of impaired vs. non-impaired: 18 (13.5–24) vs. 9 (6.0–14); *p* = 0.0004], while scores of anxiety were not significantly different between the two groups.

### Association between cognitive impairment of each subdomain and motor or non-motor symptoms in patients with de novo PD

After adjusting for age, sex, and disease duration, multivariable logistic regression analyses for independent motor or non-motor symptoms were conducted for impairment in each cognitive subdomain (Table 3).

**Table 3:** Adjusted odds ratios for cognitive impairment of subdomains in patients with de novo PD.

Subdomain	Variables	Odds ratio*	Confidence interval	P value	Adjusted P value^$^
Attention	UPDRS-III	n.a	n.a	n.a	n.a
	HY stage	n.a	n.a	n.a	n.a
	BDI	n.a	n.a	n.a	n.a
	BAI	n.a	n.a	n.a	n.a
	PFS	n.a	n.a	n.a	n.a
	SCOPA-AUT	n.a	n.a	n.a	n.a
Language	UPDRS-III	1.0668	0.9905–1.1489	0.0877	0.3508
	HY stage	4.7496	1.0074–22.3927	**0.0489**	0.1956
	BDI	1.2680	0.6774–2.3738	0.4579	1.0000
	BAI	1.0558	0.9646–1.1557	0.2387	0.9548
	PFS	1.0053	0.9626–1.0499	0.8119	1.0000
	SCOPA-AUT	1.0526	0.9687–1.1437	0.2262	0.9048
Memory	UPDRS-III	1.0687	1.0087–1.1322	**0.0242**	0.0968
	HY stage	9.136	1.9618–42.3363	**0.0048**	**0.0192**
	BDI	1.0916	1.0063 -1.1843	**0.0349**	0.1396
	BAI	0.9971	0.9216–1.0788	0.9424	1.0000
	PFS	1.0409	0.6271–1.7278	0.8767	1.0000
	SCOPA-AUT	1.1726	1.0650–1.2711	**0.0008**	**0.0032**
Visuospatial	UPDRS-III	1.0970	1.0225–1.1770	**0.0099**	**0.0396**
	HY stage	7.9772	1.4522–43.8207	**0.0169**	0.0676
	BDI	1.0779	1.0005–1.1614	**0.0486**	0.1944
	BAI	1.0736	0.9679–1.1908	0.1795	0.7180
	PFS	1.0299	0.9901–1.0712	0.1426	0.5704
	SCOPA-AUT	1.0654	0.99–1.1464	0.0906	0.3624
Executive	UPDRS-III	1.0651	1.0034–1.1306	**0.0383**	0.1532
	HY stage	5.7182	1.281–25.5247	**0.0223**	0.0892
	BDI	1.0924	1.0113–1.1801	**0.0247**	0.0988
	BAI	1.0185	0.9283–1.1174	0.6988	1.0000
	PFS	1.0484	1.0079–1.0905	**0.0187**	0.0748
	SCOPA-AUT	1.1252	1.0383–1.2193	**0.0040**	**0.0160**

*Odds ratio was obtained after adjusting for age, sex, and disease duration.

^$^P-value adjustment with Bonferroni correction was conducted for multiple comparisons.

PD, Parkinson’s disease; UPDRS-III, Unified Parkinson’s disease rating scale-part 3; BDI, Beck depression inventory; BAI, Beck anxiety inventory; PFS, Parkinson’s disease fatigue scale; SCOPA-AUT, the Scale for Outcomes in Parkinson’s disease-Autonomic questionnaire.

Significant values are in bold.

Specifically, language impairment was associated with higher HY stage scale (OR = 4.7496, 95% confidence interval [CI] = 1.0074–22.3927, *p* = 0.0489). Memory impairment was related to higher motor scores of the UPDRS-III (OR = 0.0242, 95% CI = 1.0087–1.1322, *p* = 0.0242) and the HY stage (OR = 9.136, 95% CI = 1.9618–42.3363, *p* = 0.0048), higher depression scores (OR = 1.0916, 95% CI = 1.0063 -1.1843, *p* = 0.0349), and higher autonomic scores of SCOPA-AUT (OR = 1.1726, 95% CI = 1.0650–1.2711, *p* = 0.0008), respectively. Visuospatial impairment was associated with higher scores of the UPDRS-III (OR = 1.0970, 95% CI = 1.0225–1.1770, *p* = 0.0099) and the HY stage (OR = 7.9772, 95% CI = 1.4522–43.8207, *p* = 0.0169), and higher depression scores (OR = 1.0779, 95% CI = 1.0005–1.1614, *p* = 0.0486), respectively. Executive impairment was related to higher scores of the UPDRS-III (OR = 1.0651, 95% CI = 1.0034–1.1306, *p* = 0.0383) and the HY stage (OR = 5.7182, 95% CI = 1.281–25.5247, *p* = 0.0223), higher depression scores (OR = 1.0924, 95% CI = 1.0113–1.1801, *p* = 0.0247), higher fatigue scores (OR = 1.0484, 95% CI = 1.0079–1.0905, *p* = 0.0187), and higher autonomic scores of SCOPA-AUT (OR = 1.1252, 95% CI = 1.0383–1.2193, *p* = 0.0040), respectively.

In addition, post-hoc multiple comparisons with Bonferroni correction (Table 3, the rightmost column) showed significant relationships between each cognitive domain and clinical symptoms. Memory impairment was associated with not only HY stage (*p* = 0.0192) but also SCOPA-AUT (*p* = 0.0032). Visuospatial dysfunction was related to UPDRS-III (*p* = 0.0396), and executive impairment was linked to SCOPA-AUT (*p* = 0.0160). However, language dysfunction was not associated with any symptoms.

## Discussion

Cognitive decline significantly impacts health-related quality of life in patients with PD. MCI is a high-risk factor for the development of dementia in patients with PD. Thus, early suspicion or detection of cognitive impairment in patients with PD is important for proper management of PD. The reported prevalence of MCI in patients with PD ranges from 15 to 70% and the frequency of MCI in patients with de novo PD ranges from 15 to 50%^8–10^. In the present study, among all the patients with newly diagnosed PD, 56.7% had MCI, which was slightly higher than those reported in the literature. Such difference might be due to different demographics. Our study population was older and less educated. Both old age and less education could be factors contributing to the development of MCI more prevalently in our study population. Moreover, young-aged people diagnosed with de novo PD in our hospital were reluctant to undergo a neuropsychological test at the initial evaluation compared to old-aged patients with de novo PD. In addition, we applied one standard deviation (SD) below the mean to determine impairment in different domains. In the literature, it is more common to use 1.5 SD deviation below the mean rather than 1 SD deviation. Therefore, such a relatively high cut-off value of 1 SD might among the possible reasons for the rather high MCI prevalence in our study population.

Compared with the non-PD-MCI group, we found that the PD-MCI group showed higher scores of motor severity, including rigidity score, total motor score, and HY stage, in line with the literature^8,11^. In addition, the bradykinesia score exhibited a tendency of association with PD-MCI, although such association was not statistically significant (*p* = 0.056 in Table 1). Besides, motor symptoms were widely associated with most cognitive domains except the attention domain (Fig. 1). Such a relationship between motor symptoms and MCI in patients with de novo PD is expected. As PD progresses, dopaminergic and non-dopaminergic neurodegenerations continuously occur from the viewpoint of the chronic progression of neurodegenerative disorders^12^. Braak et al. have previously demonstrated that cortical Lewy bodies are implicated in the pathological progression of PD^13^. Taken together, our results were in line with the general concept that MCI inevitably is an indicator of PD progression, since the MCI group with PD showed higher scores of motor symptoms and HY stage compared with the non-MCI group with PD.

For the association of MCI with various non-motor symptoms in patients with de novo PD, we found that MCI in de novo PD state was related to depression and autonomic dysfunction (more specifically, GI dysautonomia), but not to anxiety or fatigue symptom (Table 2). Furthermore, we demonstrated a detailed relationship between each cognitive deficit and depression or dysautonomia in patients with de novo PD (Table 3 and Fig. 1). Our results provide clinical evidence for a specific link between cognitive impairment and depression or dysautonomia in drug-naïve de novo PD state.

Depression is common in patients with any stage of PD. Its relationship with cognitive dysfunction has been widely studied, showing a significant association^4^. For instance, Jones and colleagues showed that cognitive decline is associated with depressive symptom, but not with apathy or anxiety in a 4-year follow-up study from a cohort with de novo PD^14^. Likewise, the present study revealed that patients with de novo PD with MCI exhibited higher scores of depression, but not of anxiety and fatigue than patients with de novo PD without MCI (Table 2). We also found that both frontal-executive and visuospatial dysfunctions were closely related to depression in patients with de novo PD (Table 3 and Fig. 1). Our results imply that not only the prefrontal dopaminergic pathways, but also the posterior cortical non-dopaminergic pathways are significantly implicated in the pathogenesis of depression in PD^6,7^. In other words, our findings suggest that depression might be an important indicator of impaired cognition in the early stages of PD. Similarly, some studies have shown that depression could be a risk factor for MCI in patients with PD^14,15^. However, whether depression is related to the development of PD-dementia remains unclear^16,17^. Research on the clinical implications of depression in relatively advanced stages of PD is required to address this issue. In addition, depression is known to be associated with cognitive progression in patients with Alzheimer’s disease^18^, inferring that it is widely involved in the pathogenesis of cognitive decline in various neurodegenerative disorders beyond PD.

One main aim of the current study was to determine the association of each cognitive domain with specific motor or non-motor symptom considering the “dual syndrome hypothesis” of PD as introduced previously^6,7^. Frontal-executive dysfunction implies nigrostriatal dopaminergic degeneration, while memory and/or visuospatial dysfunction reflects non-dopaminergic degenerations involving posterior cortical areas of the brain^19^. However, contrary to our expectations, our results failed to reveal the characteristic linkage between different cognitive domains and specific motor or non-motor symptoms in our study population. Instead, we found that motor symptoms of UPDRS and HY stage were widely associated with cognitive deficits except for attention. This implies that dopaminergic degenerations are widely implicated in cognitive functions even in the early stages of PD. Moreover, our results showed that only fatigue symptoms were linked to frontal-executive impairment, suggesting that fatigue might be selectively implicated in the frontostriatal cognitive loop^20^. Besides, relatively different patterns of the association of non-motor symptoms with cognitive deficits were noted in patients with de novo PD. Memory impairment was associated with depression and dysautonomia, but not with anxiety and fatigue. While visuospatial dysfunction was related only to depression, frontal-executive dysfunction was more broadly associated with depression, fatigue, and dysautonomia, but not with anxiety. Collectively, it is reasonable to infer that the most relevant non-motor symptom linked to cognitive impairment in de novo PD is depression, followed by dysautonomia, and fatigue, whereas anxiety seems to be independent of cognitive impairment. Therefore, clinicians need to check autonomic dysfunction and/or depressive mood if a patient with PD has MCI. In addition, being depressed can lead to worse performance on cognitive tests in patients with PD, thereby close observations and appropriate managements are necessary in patients with PD, especially with both depression and MCI.

In the present study, we examined the relationship between MCI and various autonomic dysfunctions in de novo patients with PD in detail (Table 2, the bottom part). We demonstrated that diverse domains of dysautonomia were significantly linked to MCI in patients with PD. Similarly, previous studies have demonstrated that CV dysautonomia including orthostatic hypotension might be a risk factor for cognitive decline in patients with PD^21^. Notably, we found that GI dysautonomia was significantly related to PD-MCI. Jones and other colleagues have recently reported that GI symptoms are uniquely associated with cognitive impairment from a PD cohort^22^. However, the accurate pathomechanism and the causal relationship between cognitive impairment and GI symptoms have not been well identified yet. Some researchers have suggested that gut health associated with microbiota might be connected to the cognitive status of patients with PD^23^. Others have provided a concept that cholinergic degeneration in the peripheral GI tract and central cholinergic systems might be highly related to each other and play a role in the development of not only cognitive decline, but also constipation in patients with PD^24^.

In general, multiple comparison adjustments in a logistic regression analysis with a relatively small number of participants might miss the real significant associations. Therefore, in this pilot study, we supposed that it is more desirable to interpret the multivariable logistic regression analysis with a basic analysis (Table 3, p-values) rather than multiple comparison tests with Bonferroni correction (Table 3, adjusted p-values), although type I error may increase. Interestingly, the multiple comparison tests revealed that autonomic dysfunction was significantly related to memory and executive impairments. Therefore, further studies with large numbers are needed.

When interpreting the results of this study, care is needed because of its several shortcomings. First, the interpretation of our results needs to be done carefully because of the limitation of a retrospectively designed cross-sectional study. This type of study is prone to contain selection bias. Second, the number of patients was not large. Thus, further statical analyses in detail were limited for our study population. Nevertheless, we found some clinical implications for the relationship between cognitive dysfunctions and motor or non-motor symptoms in patients with de novo PD. Third, some of our patients with PD might have a misdiagnosis since this study was only for de novo PD in the early stages of the disease. However, strict inclusion and exclusion criteria were applied in this study to overcome this weakness.

In conclusion, we found a characteristic relationship between cognitive dysfunctions and motor or non-motor symptoms in patients with de novo PD. Motor symptoms were widely associated with cognitive deficits in our study population. On the other hand, non-motor symptoms showed different patterns of cognitive impairments. Especially, dysautonomia and depression were significantly related to cognitive deficits in visuospatial and frontal-executive domains. Further studies are warranted to confirm our findings on the pathogenesis of cognitive impairment in the very early stages of PD.

## Materials and methods

### Patients

Patients clinically diagnosed with drug naïve PD according to the UK Brain Bank criteria^25^ who were registered in our movement disorders clinic from July 2017 to March 2021 were evaluated. All the patients underwent brain magnetic resonance imaging (MRI) to identify brain lesions and dopamine transporter (DAT) imaging to confirm the diagnosis of PD^26^. To exclude atypical parkinsonism and secondary parkinsonism including normal pressure hydrocephalus, patients showing any considerable ischemic burden or ventricular enlargement were excluded. In addition, any patients presenting with atypical features including recurrent falls, poor levodopa responsiveness, or significant dysautonomia were excluded. Patients having any serious medical problems were excluded because such problems might have a significant impact on the motor or non-motor symptoms beyond PD^27^. To meet the purpose of the current study, patients who did not undergo a neuropsychological test were also excluded.

Out of a total of 119 patients registered in our movement disorder clinic, 60 subjects with drug naïve de novo PD were finally enrolled for this study. All of them exhibited a significant improvement in anti-parkinsonian treatments during 2.9 ± 0.9 (mean ± SD) years of follow-ups. We excluded the remaining 59 people; 37 patients with PD were not in de novo state, 5 had multiple system atrophy, 4 had vascular parkinsonism, 3 had normal pressure hydrocephalus, 3 had unspecified parkinsonism, 2 had progressive supranuclear palsy, 2 had essential tremor, 2 had drug-induced parkinsonism, and 1 had dementia with Lewy bodies.

### Clinical assessments

In our movement disorder clinic, patients with parkinsonism are routinely evaluated for clinical details at the time of registration. The Unified Parkinson’s Disease Rating Scale (UPDRS) part III and the Hoehn and Yahr (HY) stage were used to assess motor symptoms^27^. Cardinal motor subscores (such as tremor, rigidity, and bradykinesia) and motor subtypes of tremor-dominant/intermediate/postural instability gait difficulty (PIGD) were evaluated according to the literature^27,28^. To assess NMSs, the Korean version of the Beck Depression Inventory (BDI)^29^ for depressive symptoms, the Korean version of the Beck Anxiety Inventory (BAI)^30^ for anxious mood, the Parkinson’s Disease Fatigue Scale (PFS)^31,32^ for fatigue symptom, and the Korean version of the Scale for Outcomes in Parkinson's Disease-Autonomic (SCOPA-AUT)^33^ for various autonomic dysfunctions were used.

The Seoul Neuropsychological Screening Battery-II (SNSB-II), a comprehensive neuropsychological test for assessing attention and working memory, language, memory, visuospatial, and frontal/executive domains was performed for all the subjects^34^. The percentile score in each cognitive test was adjusted by age and years of education from the norm established for the Korean population^35^. According to the movement disorder society task force guideline^36^, cognitive domains in the Korean version of representative tests are selected as follows: (1) attention and working memory (digit span forward, digit span backward, and Stroop color-word test), (2) language function (Boston Naming Test), (3) memory function (Seoul Verbal Learning Test-delayed recalls/recognition and Rey Complex Figure Test-delayed recalls/recognition), (4) visuospatial function (Rey Complex Figure Test and clock drawing test), and (5) executive function (Controlled Oral Word Association Test—animal/phonemic, Digit Symbol Coding, and part B of the Trail Making Test). The cognitive impairment of each neuropsychological test was determined by 1 standard deviation below appropriate norms. MCI in patients with PD was defined according to level II criteria of the movement disorder society guideline except for the language function test^36^. Briefly, MCI was defined with either two impaired tests for one cognitive domain or one impaired test for two or more different cognitive domains. The composite score in each cognitive domain was calculated from the percentiles of each cognitive test. Cognitive impairment of each of the 5 domains was assessed based on the composite percentile score of cognitive tests in the SNSB, as described above.

### Statistics

For two-group comparisons, the Chi-square test for categorical variables and the student’s t-test or the Mann–Whitney *U* test for continuous variables were used after normality tests. The Shapiro-Wilks test for normality was used. To uncover the association between cognitive impairment of subdomains and motor or non-motor symptoms in patients with de novo PD, we conducted multivariable logistic regression analysis for UPDRS-III score, HY staging scale, depression score, anxiety score, fatigue score, and SCOPA-AUT score as independent variables, after controlling for age, sex, and disease duration. All the statistical analyses were performed using Rex version 3.6.3 (URL http://rexsoft.org).

### Ethical Approval

All procedures performed in studies involving human participants were in accordance with the ethical standards of the institutional and/or national research committee and with the 1964 Helsinki declaration and its later amendments or comparable ethical standards. The ethics committee of our Institutional Review Board approved this retrospectively designed study (Approval No. 2021–03-035) with a waiver of informed consent.

## Supplementary Information

Supplementary Information 1.

Supplementary Information 2.

## Data Availability

The datasets generated and analyzed during the current study are available from the corresponding author on reasonable request.
