# Supplementary figures and images for: Associations of cognitive dysfunction with motor and non-motor symptoms in patients with de novo Parkinson’s disease
Source: Sci Rep. 2022 Jul 6;12:11461. doi: 10.1038/s41598-022-15630-8 (PMC9259652; doi:10.1038/s41598-022-15630-8)

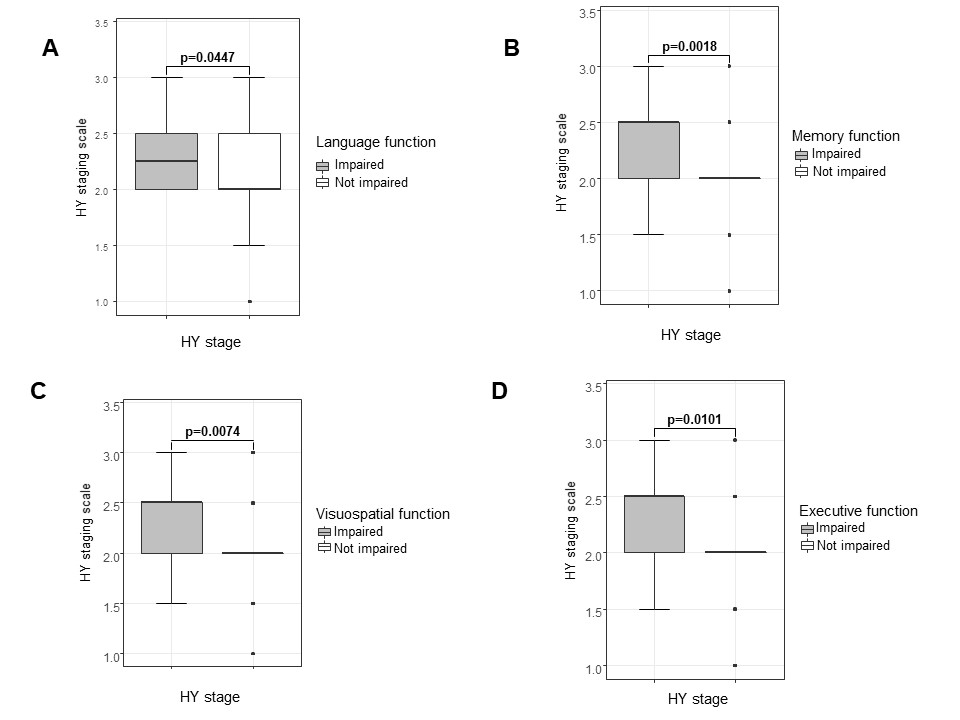

Supplement: Supplementary file 1 — Supplementary Information 1. [file 41598_2022_15630_MOESM1_ESM.jpg]
