# Supplementary material for: Associations of cognitive dysfunction with motor and non-motor symptoms in patients with de novo Parkinson’s disease
Source: Sci Rep. 2022 Jul 6;12:11461. doi: 10.1038/s41598-022-15630-8 (PMC9259652; doi:10.1038/s41598-022-15630-8)
Supplement: Supplementary file 2 — Supplementary Information 2. [file 41598_2022_15630_MOESM2_ESM.docx]

The Supplementary Information file.

**Associations of cognitive dysfunction with motor and non-motor symptoms in patients with *de novo* Parkinson’s disease**

Kyum-Yil Kwon^*^, Suyeon Park, Rae On Kim, Eun Ji Lee, Mina Lee

Supplementary Table 1. Comparison of motor or non-motor symptoms between de novo parkinsonian patients with and without impairment in each cognitive domain

| Cognitive subdomain | Variables | Impaired,  median (IQR) | Non-impaired  Median (IQR) | P value |
| --- | --- | --- | --- | --- |
| Attention | UPDRS-III | n.a. | 26.5 (16.75 – 31.25) | n.a. |
|  | HY stage | n.a. | 2 (2 – 2.5) | n.a. |
|  | BDI | n.a. | 9 (3.75 – 13.50) | n.a. |
|  | BAI | n.a. | 6 (2 – 11) | n.a. |
|  | PFS | n.a. | 43 (28.5 – 54.25) | n.a. |
|  | SCOPA-AUT | n.a. | 12.5 (7.75 – 18.25) | n.a. |
| Language | UPDRS-III | 31.5 (27.0 – 36.25) | 25 (14.5 – 30.25) | **0.0291** |
|  | HY stage | 2.25 (2 – 2.5) | 2 (2 – 2.5) | **0.0447** |
|  | BDI | 12.0 (8.25 – 15.25) | 7.5 (3.0 – 12.25) | 0.2111 |
|  | BAI | 7.0 (6.0 – 8.25) | 4.5 (1.75 – 11.0) | 0.1129 |
|  | PFS | 44.5 (33.0 – 61) | 42.5 (28.5 – 54) | 0.4374 |
|  | SCOPA-AUT | 17.5 (8.75 – 22.25) | 12.0 (7.0 – 18.0) | 0.0995 |
| Memory | UPDRS-III | 30 (19.5 – 38) | 24 (13.0 – 29) | **0.0299** |
|  | HY stage | 2.5 (2 – 2.5) | 2.0 (2 – 2.0) | **0.0018** |
|  | BDI | 10 (6 – 17) | 6 (3 – 12) | 0.0763 |
|  | BAI | 6 (2.5 – 11) | 5 (2.0 – 9) | 0.6494 |
|  | PFS | 44 (30.5 – 61) | 41 (27.0 – 53) | 0.2402 |
|  | SCOPA-AUT | 18 (13 – 23) | 8 (6 – 13) | **<0.0001** |
| Visuospatial | UPDRS-III | 31 (27.5 – 37.5) | 24 (15.0 – 30.0) | **0.0117** |
|  | HY stage | 2.5 (2 – 2.5) | 2.0 (2 – 2.0) | **0.0074** |
|  | BDI | 16 (7.5 – 21) | 7 (3.0 – 12) | **0.031** |
|  | BAI | 7 (5 – 12.5) | 5 (2 – 9.0) | 0.0608 |
|  | PFS | 50 (29.5 – 64) | 42 (29.0 – 53) | 0.1538 |
|  | SCOPA-AUT | 15 (11 – 23) | 11 (7 – 18) | **0.0262** |
| Executive | UPDRS-III | 30 (24.5 – 37.5) | 24 (16.0 – 31.0) | **0.0351** |
|  | HY stage | 2.5 (2 – 2.5) | 2.0 (2 – 2.0) | **0.0101** |
|  | BDI | 12 (9 – 19.5) | 6 (3 – 12.0) | **0.0096** |
|  | BAI | 7 (3 – 11) | 5 (2 – 11) | 0.5134 |
|  | PFS | 51 (33.5 – 64) | 41 (27.0 – 50) | **0.023** |
|  | SCOPA-AUT | 18 (13.5 – 24) | 9 (6.0 – 14) | **0.0004** |

Data are shown as median (interquartile range)

IQR, interquartile range; UPDRS, Unified Parkinson’s disease rating scale; BDI, Beck depression inventory; BAI, Beck anxiety inventory; PFS, Parkinson’s disease fatigue scale; SCOPA-AUT, the Scale for Outcomes in Parkinson’s disease-Autonomic questionnaire

Supplementary Figure 1. **Comparison of HY stage between patients with de novo PD with and without impairment in each cognitive domain.**

Comparison of HY stage between patients with *de novo* PD with and without language impairment (A). Comparison of HY stage between those with and without memory impairment (B). Comparison of HY stage between those with and without visuospatial impairment (C). Comparison of HY stage between those with and without executive impairment (D).

Abbreviations: HY stage, Hoehn and Yahr stage; PD, Parkinson’s disease.
